# Author Correction: Extreme intratumour heterogeneity and driver evolution in mismatch repair deficient gastro-oesophageal cancer

**DOI:** 10.1038/s41467-020-14602-8

**Published:** 2020-01-29

**Authors:** Katharina von Loga, Andrew Woolston, Marco Punta, Louise J. Barber, Beatrice Griffiths, Maria Semiannikova, Georgia Spain, Benjamin Challoner, Kerry Fenwick, Ronald Simon, Andreas Marx, Guido Sauter, Stefano Lise, Nik Matthews, Marco Gerlinger

**Affiliations:** 1 Translational Oncogenomics Laboratory, Centre for Evolution and Cancer, The Institute of Cancer Research, London, SW3 6JB United Kingdom; 2 Bioinformatics Core, Centre for Evolution and Cancer, The Institute of Cancer Research, London, SM2 5NG United Kingdom; 3 Tumour Profiling Unit, The Institute of Cancer Research, London, SW3 6JB United Kingdom; 4 Institute of Pathology, University Medical Center Hamburg-Eppendorf, 20246 Hamburg, Germany; 5 Gastrointestinal Cancer Unit, The Royal Marsden Hospital, London, SW3 6JJ United Kingdom; 6 Present Address: Biomedical Research Centre, The Royal Marsden Hospital, London, SM2 5PT United Kingdom; 7 Present Address: Institute of Pathology, University Hospital Fuerth, 90766 Fuerth, Germany

**Keywords:** Cancer, Cancer genetics, Cancer genomics, Gastrointestinal cancer, Tumour heterogeneity

Correction to: *Nature Communications* 10.1038/s41467-019-13915-7, published online 16 January 2020.

The original version of this Article omitted the following funding source from the Acknowledgements:

The study was supported by grants from the Schottlander Research Charitable Trust.

This has been corrected in the PDF and HTML versions of the Article.

