# Supplementary material for: Extreme intratumour heterogeneity and driver evolution in mismatch repair deficient gastro-oesophageal cancer
Source: Nat Commun. 2020 Jan 16;11:139. doi: 10.1038/s41467-019-13915-7 (PMC6965135; doi:10.1038/s41467-019-13915-7)
Supplement: Supplementary file 3 — Description of Additional Supplementary Files [file 41467_2019_13915_MOESM3_ESM.pdf]

Description of Additional Supplementary Files

File Name: Supplementary Data 1

Description: Mseq mutations calls and depths.

File Name: Supplementary Data 2

Description: Putative driver mutations.

File Name: Supplementary Data 3

Description: HLA mutation calls and LOH analysis.
